# Shoulder Pain and Disability Scores and the Factors Influencing Them among Orthopedic Surgeons Working in the Kingdom of Saudi Arabia: A Cross-Sectional Study

**DOI:** 10.3390/jpm14010055

**Published:** 2023-12-30

**Authors:** Faya Ali Asiri, Abdulrhman Abdullh Alqhtani, Abdullah Hassan Assiri, Mohammed Hassan Alqahtani, Dhuha Saeed Motlag, Jaya Shanker Tedla, Saad Ali Alwadai

**Affiliations:** 1 Department of Orthopedics, Ahad Rufaidah General Hospital, Abha 62242, Saudi Arabia; faasiri@moh.gov.sa; 2 Department of Orthopedics, Aseer Central Hospital, Abha 62523, Saudi Arabia; ababalqhtani@moh.gov.sa (A.A.A.); aassiri58@moh.gov.sa (A.H.A.); malqahtani286@moh.gov.sa (M.H.A.); dmotlag@moh.gov.sa (D.S.M.); saaalwadai@moh.gov.sa (S.A.A.); 3 Department of Medical Rehabilitation Science, College of Applied Medical Sciences, King Khalid University, Abha 62421, Saudi Arabia

**Keywords:** shoulder pain, disability, orthopedic surgeons

## Abstract

(1) Background: Musculoskeletal pain is common among orthopedic surgeons. Their common musculoskeletal issues include shoulder pain and disability. Many associated factors could lead to this pain and disability; by identifying these, we can prevent orthopedic surgeons’ pain and improve their functional capacity. (2) Methods: This study aimed to gather quantitative data regarding the shoulder pain and disability experienced by orthopedic surgeons. It also aimed to explore the potential correlations between demographic characteristics and work-related factors and their pain and disability. This study interviewed 150 orthopedic surgeons working in the Kingdom of Saudi Arabia, asking questions on the Shoulder Pain and Disability Index (SPADI) scale and about their demographic characteristics. (3) Results: All interviewed orthopedic surgeons were male, married, and nonsmokers. Their SPADI pain subsection score was 25.24%, their SPADI disability subsection score was 21.25%, and their total SPADI score was 22.79%. Among the examined demographic characteristics, total SPADI scores have a significant positive correlation with body weight (Spearman’s *ρ* = 0.432; *p* < 0.05) and body mass index (BMI; *ρ* = 0.349; *p* < 0.05). (4) Conclusions: Our findings indicate that all orthopedic surgeons generally suffer from moderate shoulder pain and disability. Body weight and body mass index are important factors that may influence shoulder pain and disability among orthopedic surgeons.

## 1. Introduction

Orthopedic surgeons’ rate of shoulder dysfunction might differ due to various factors, such as age, surgical specialization, volume and type of procedures performed, and ergonomic practices [1]. Due to the physically demanding nature of their profession, which frequently entails long hours of standing, wearing heavy lead aprons during fluoroscopy, and performing repeated activities in uncomfortable postures, orthopedic surgeons are susceptible to acquiring musculoskeletal issues, including shoulder discomfort [2].

Several studies have examined the frequency of musculoskeletal issues, such as shoulder dysfunction, among orthopedic surgeons, finding that this group experiences shoulder problems at various rates (20–80%). Variations in research design, participant characteristics, and shoulder dysfunction classifications can potentially explain the considerable variation in these estimates [3,4,5]. Shoulder impingement, tendinitis, bursitis, and rotator cuff ailments are common issues among surgeons. Overuse, improper ergonomic techniques, and repeated stress on the shoulder joint and surrounding tissues can all cause these disorders [6].

To reduce the risk of shoulder dysfunction, orthopedic surgeons may implement ergonomic strategies such as appropriate body mechanics, ergonomic apparatus, and routine exercise and stretching [4]. However, the prevalence of shoulder dysfunction remains a concern in this field, and efforts are ongoing to enhance workplace ergonomics and reduce the risk of musculoskeletal problems among orthopedic surgeons [1].

Shoulder pain and dysfunction can significantly affect orthopedic surgeons’ personal lives and the quality of treatment they provide. Shoulder problems can impact orthopedic surgeons in different ways. For example, shoulder pain and dysfunction can result in persistent distress and pain, making it difficult for orthopedic surgeons to perform their daily duties inside and outside of the operating room and negatively impacting their quality of life and well-being. In addition to discomfort, shoulder disorders can reduce the affected shoulder’s range of motion and mobility, hindering their ability to perform surgery effectively and affecting their daily lives [7].

During surgical procedures, orthopedic surgeons require high accuracy and manual dexterity. Shoulder discomfort or dysfunction can result in tremors and diminished dexterity, and make it challenging to maintain the necessary degree of accuracy during surgical procedures. These effects may impact the overall success of surgical procedures. In addition, managing persistent shoulder discomfort can cause significant mental and physical fatigue. Surgeons may encounter heightened weariness levels, potentially exacerbating their long-term risk of burnout. Burnout can potentially harm healthcare professionals’ capacity to deliver superior patient care and to exercise sound judgment in medical decision making [8].

Orthopedic surgeons may be compelled to decrease their surgical caseload or to contemplate early retirement due to significant shoulder complications. These decisions can result in the loss of skilled individuals within the area, impacting patient access to specialist treatment. Shoulder pain during surgery may result in feelings of discomfort or a reduced ability to concentrate, potentially jeopardizing patient safety. To achieve optimal patient outcomes, surgeons must maintain a high concentration level and execute procedures at their highest capacity. The physically demanding nature of the work performed by orthopedic surgeons places them at a heightened risk of developing shoulder difficulties. Prolonged exposure to inadequate ergonomic practices can worsen pre-existing shoulder problems and contribute to developing chronic health complications [9].

Therefore, it is crucial to determine shoulder pain and dysfunction-related issues among orthopedic surgeons in the Kingdom of Saudi Arabia. Previous research studies have found many musculoskeletal injuries among surgeons working in the Kingdom of Saudi Arabia [10,11]. While some of these have examined musculoskeletal injuries among orthopedic surgeons working in the Kingdom of Saudi Arabia [12,13,14], none have reported quantitative evidence of their shoulder pain and dysfunction. Therefore, this study aimed to obtain quantitative proof of shoulder pain and dysfunction among orthopedic surgeons in the Kingdom of Saudi Arabia and to assess the correlations between their demographic characteristics and work-related factors and their pain and dysfunction.

## 2. Materials and Methods

This cross-sectional research was approved by the King Khalid University Research Ethics Committee (HAPO-06-B-001), with acceptance number ECM#2023-2130. Orthopedic surgeons working in the Kingdom of Saudi Arabia were identified by obtaining their details from orthopedic associations, WhatsApp groups, social media, and other resources. Convenience sampling was used, and all orthopedic surgeons practicing in the Kingdom of Saudi Arabia were invited to participate, regardless of their nationality (Saudi/non-Saudi), orthopedic specialty, and rank (e.g., residents, specialists, or consultants). Orthopedic surgeons not currently practicing in the clinics who were unwilling to provide consent, had any other health-related issues, or did not provide all the essential information in the questionnaire were excluded from this study.

The study was conducted for one year from August 2022 to July 2023. Two investigators, AAA and JST, obtained contact details of potential subjects from the resources mentioned above and communicated with each one by email, WhatsApp, mobile, or telephone. The contacted orthopedic surgeons were provided with a Google form or physical form. The investigators who collected the data were unbiased in the data analysis. We contacted around 500 orthopedic surgeons, and 150 participated in the study. Hence, the response rate was about 30 percent. The remaining people who could not participate in the survey may have stopped practicing, were too busy to participate, changed their profession, or were in active administration, etc. The data collection sheet had a written consent form explaining the study details, and only those who consented proceeded to the questionnaire. The consent form assured the confidentiality of the participants’ information and collected their contact details to allow the investigators to contact them for clarification. The data collection sheet contained information about the surgeons’ demographics, such as age, height, weight, years of experience, marital status, smoking history, professional practices, and answers to the SPADI scale questions. The BMI was calculated based on the height and weight reported by the participants.

The study’s sample size was calculated using the web-based ClinCalc software (https://clincalc.com/Stats/SampleSize.aspx, accessed on 4 April 2023). This sample-size calculator determines the required number of subjects to provide adequate study power. We used the “one study group vs. the population” study-group design option and the “continuous (means)” primary-endpoint option. The anticipated mean and standard deviation (SD) of the SPADI scores of the known population were taken from the study by Almogbil et al. that was conducted on healthcare workers in the Kingdom of Saudi Arabia [15]. The estimated mean SPADI score in our study group was set to 26. The type 1 error (alpha) was established as 0.05, and the power (the ability to detect a difference between the groups when a discrepancy exists) was set to 85%. With these settings, the required study-sample size was 142. Since we expected some respondents to drop out of the study, we chose a sample size of 150 participants.

### 2.1. Outcome Measure

The SPADI scale is a widely used self-reported measure designed to evaluate the effects and consequences of shoulder pain on an individual’s everyday activities and functional abilities. This instrument was created to assist physicians and researchers in better comprehending and quantifying the physical and practical constraints associated with shoulder discomfort. The SPADI scale was specifically developed to evaluate several dimensions of shoulder pain, primarily focusing on pain severity as an indicator of the degree of shoulder discomfort. Another dimension assesses how much the shoulder discomfort affects an individual’s capacity to perform routine tasks. The SPADI scale was initially created by Roach et al. in 1991 to provide a dependable and accurate assessment of shoulder pain and impairment. It comprises 13 items, with 5 dedicated to evaluating the pain level and 8 focused on assessing disability. Each item within the SPADI scale is estimated using a numerical scale from 0 (no pain or impairment) to 10 (severe pain or disability). Pain is assessed by asking participants to evaluate their pain intensity while engaging in different activities and at rest. Disability is assessed by asking participants to rate their difficulty in completing various everyday tasks due to shoulder discomfort [16,17].

The SPADI scale is scored by summing the individual item scores, categorized into pain and disability domains. The SPADI scores range from 0 to 100, with greater scores indicating higher pain or impairment. The SPADI scale can be used by healthcare professionals to track the progression of a subject’s shoulder ailment over a period of time or to evaluate the efficacy of therapies. The SPADI scale has demonstrated favorable reliability and validity, making it a valuable instrument in clinical and research contexts. This tool has been extensively used in studies on shoulder pain and is regarded as a useful instrument for evaluating patient-reported outcomes in this domain. Therefore, the SPADI scale is a suitable instrument for healthcare practitioners and researchers to use to measure how much and individual’s shoulder discomfort affects their daily activities. It is commonly used to examine shoulder-related diseases and to monitor patients’ development [18].

After obtaining data from various sources, we screened and entered the data into the SPSS software (version 28). The descriptive variables were coded and converted into numerical values, and respondents with missing data were recontacted to complete the missing fields. Respondents with missing data after the second contact were removed from the data analysis.

### 2.2. Data Analysis

Data analyses were conducted using SPSS software (version 28). The mean, SD, minimum, and maximum were calculated for the demographic variables and SPADI scores. The level of significance, measured by the *p*-value, was set to be less than 0.05. The existence of a relationship between the variables was assessed through the Spearman’s rank correlation coefficient (*ρ*) and interpreted based on the guidelines provided by Akoglu [19]: weak, 0 ≤ |*ρ*| < 0.4; moderate, 0.4 ≤ |*ρ*| < 0.7; strong, 0.7 ≤ |*ρ*| <0.9.

## 3. Results

For this study, we contacted more than 500 orthopedic surgeons by various means, including via emails, WhatsApp messages, phone calls, and personal communications. From these efforts, we obtained data on a sample of 157 orthopedic surgeons, of which only one was female and six had incomplete details. Therefore, we decided to exclude these seven respondents from this study, leaving 150 male respondents for analysis. The participants’ mean ± SD age was 35.04 ± 8.42 years. The descriptive statistics for the participants’ height, weight, body mass index (BMI), years of experience, and work hours per day are provided in Table 1. In addition, the numbers and percentages for the participants’ responses to questions on marital status, city of residence in Saudi Arabia, nationality, smoking status, position, and subspecialty under orthopedics are provided in Table 2. Most participants were Saudi nationals, married, nonsmokers, and residents; lived in the Abha/Aseer region; and had general orthopedics as their subspecialty.

Table 3 contains descriptive statistics on the participants’ scores for each SPADI scale item and domain (pain (*n* = 5) and disability (*n* = 8)) and their total SPADI score. Their mean percentage was 25.24% for the pain domain and 21.25% for the disability domain. Their mean total SPADI score was 29.62, and their mean total SPADI percentage was 22.79%.

We explored correlations between the different demographic characteristics and the total SPADI percentages in Table 4. The total SPADI percentages correlated significantly positively with body weight (*ρ* = 0.432; *p* = 0.002) and BMI (*ρ* = 0.349; *p* = 0.013), indicating that body weight and BMI significantly affect orthopedic surgeons’ shoulder pain and disability.

## 4. Discussion

Our study uniquely focused on shoulder pain and dysfunction among orthopedic surgeons in the Kingdom of Saudi Arabia. While Saudi hospitals have excellent infrastructure and an excellent workforce, shoulder pain and dysfunction are common among orthopedic surgeons working in the Kingdom of Saudi Arabia.

Mohrej et al. conducted a cross-sectional study on musculoskeletal disorders among orthopedic surgeons working in the Kingdom of Saudi Arabia. They found that shoulder/elbow-related pain among orthopedic surgeons is the third most-common problem after low back pain and neck pain. They assessed pain on a 10-point visual analog scale, with most participants having mild pain, with scores of 1–3. Our findings are similar, with a mean SPADI pain score of 25.24 out of 100 [14].

Mcquivey et al. found that musculoskeletal pain was highest among orthopedic residents and arthroplasty surgeons. They reported that 97% of their participants had procedural-related musculoskeletal pain, with a mean pain score of 3.7 out of 10, slightly higher than in our study. These musculoskeletal pains affected their stamina, concentration, degree of irritability, and other burnout symptoms, highlighting the importance of surgical ergonomics to prevent the suffering of surgeons [20,21].

A recent study in India conducted on doctors and nursing faculty found a similar relationship between obesity and pain [22]. A 19-year cohort study involving 938 women also established a positive relationship between increased BMI and musculoskeletal pain [23]. Like previous studies that reported a positive relationship between obesity and pain [24,25], our research also found a positive significant correlation between pain and body weight. According to some hypotheses, increasing body weight decreases flexibility and causes undue loading on the axial body, leading to increased pain levels when these structures are used excessively [26,27].

Almogbil et al. investigated the Kingdom of Saudi Arabia to examine the prevalence of neck and shoulder discomfort among healthcare professionals. An evaluation was conducted on a total of 409 healthcare providers, with the bulk of them being doctors. The researchers employed the SPADI scale to gauge the level of shoulder pain and disability, whereas the neck Bournemouth questionnaire was utilized to evaluate neck discomfort. They reported an average mean ± SD SPADI score of 31.8 ± 23.1, which is about 31.8%, and the current study also observed comparable overall scores among orthopedic physicians. Our study similarly observed that overweight professionals had higher SPADI scores.

To address the issues associated with shoulder pain and impairment and to uphold the standard of care offered to their patients, orthopedic surgeons should consider the following factors. Despite being physicians and surgeons themselves, it is advisable for them to promptly seek medical assessment and treatment for shoulder disorders to manage discomfort and dysfunction effectively. In addition, they should incorporate ergonomic strategies and use specialized tools to mitigate the likelihood of additional harm. Moreover, they must contemplate altering surgical methods or adopting ergonomic advancements to sustain the delivery of superior healthcare services. Orthopedic surgeons should give precedence to self-care practices, such as physical therapy, exercise, and healthy lifestyle choices, to treat and prevent shoulder issues effectively. Furthermore, they should acknowledge the significance of maintaining a work–life balance to mitigate burnout and to preserve a long-term professional trajectory [7,8,14,20,21]. The timely and proactive management of shoulder discomfort and dysfunction is paramount for orthopedic surgeons since it directly impacts their well-being and the quality of treatment they deliver. Establishing efficient communication with co-workers and healthcare experts might be advantageous to appropriately addressing these problems.

Our research observed a moderate positive correlation between body weight and BMI on SPADI scores among orthopedic surgeons. Hence, it is clear that decreasing body weight may reduce the chances of shoulder pain and dysfunction. As a commonly known factor, as people become older and busier in their profession, they become prone to accumulating weight year by year [28,29]. Similarly, if orthopedic professionals continue to become more engaged in their profession and their passion for becoming rich and famous masks the importance of health, they will slowly be prone to obesity [30,31]. Meanwhile, repeated stress on the shoulder due to continuous investigations and surgeries will cause structural and functional compromise that leads to damage and pain. Inefficient ergonomic methods and a lack of proper knowledge regarding the kinetics of their movements may lead to abnormal loads on the structures. Lack of exercise, combined with all the above factors, will increase the chance of damage.

As per our suggestions for the orthopedic surgeons, simple strategies like controlling body weight growth, decreasing their BMI by exercise and diet, following the proper mechanics during their orthopedic procedures, performing muscle-strengthening exercises at least 3–4 times per week, understanding the mechanics of pain in the initial stages, consulting with therapists specialized in ergonomic analysis, correcting abnormal procedures, and taking the treatments required to heal the affected structures at the beginning of the problems can efficiently decrease the pain and disability levels.

Investigators need to consider multiple constraints when evaluating the results of cross-sectional studies. Certain notable disadvantages associated with these kinds of research are the absence of a temporal sequence and data collection at a singular moment, which hinders the establishment of cause-and-effect relationships. Additionally, tracking changes within individuals or populations over time is not possible, and there is a potential for selection bias due to the specific point in time at which participants are chosen. Researchers should carefully evaluate these limitations in comparison with the benefits of cross-sectional designs and consider the particular research topic and context when selecting a study design. Cross-sectional studies are useful for formulating hypotheses and providing an overview of the frequency of circumstances.

Investigators should be cognizant of the many constraints associated with relying on individuals’ data. These constraints can impact the precision and dependability of the gathered data. Several notable constraints include several things. For example, respondents may exhibit social desirability bias, wherein they offer responses that align with societal norms or expectations rather than accurately representing their ideas, feelings, or actions. Linguistic hurdles and communication obstacles might result in misinterpreting a given question. Furthermore, the information provided may be unverifiable. Lastly, the participants’ moods and settings significantly influence the correctness of their replies. Notwithstanding these constraints, self-reported data can yield useful insights when employed judiciously and when complemented by other research methodologies. Researchers must provide transparency on the inherent biases and limits of self-reported measures. Additionally, they should contemplate using different data sources to triangulate their findings.

### Limitations and Future Suggestions

Our study focused only on shoulder pain and disability levels among orthopedic surgeons. Due to the cultural constraints and the lack of availability of female orthopedic surgeons in the Kingdom of Saudi Arabia, the study sample consisted only of males and did not include females. Those it included most lived in Abha, and most were residents rather than practicing orthopedic surgeons. Therefore, future studies should aim to examine cohorts with equal representation by seniority, gender, and region of Saudi Arabia to provide a clearer picture of this problem.

## 5. Conclusions

The 150 male orthopedic surgeons in the Kingdom of Saudi Arabia assessed for shoulder pain and impairment using the SPADI scale had a mean total SPADI percentage of 22.79%. The SPADI shoulder pain and disability domains had similar percentages of 20–25%. Notably, the surgeon’s body weight was significantly positively correlated with their total SPADI score (*ρ* = 0.432; *p* = 0.002).

## Figures and Tables

**Table 1:** Demographic variables of the included subjects.

Demographic Characteristic	Mean	Standard Deviation	Minimum	Maximum
Age in years	35.04	8.42	25.00	70.00
Height in meters	1.73	0.08	1.51	1.87
Weight in kilograms	84.74	18.07	46.00	128.00
BMI in kilogram/meter square	28.30	5.33	19.14	43.29
Years of experience	8.14	7.88	1	36
Hours of work/day	8.34	1.30	5.00	12.00

Note: BMI: Body Mass Index.

**Table 2:** The number of participants in each subcategory under specific variables and their percentages.

Variable	Subcategory	Frequency	Percentage (%)
Marital Status	Single	42	28
	Married	108	72
City of Residence in Saudi Arabia	Asir/Abha	81	54
	Riyadh	36	24
	Jeddah/Makkah	15	10
	Dammam/Alkhobar	18	12
Nationality	Saudi	117	78
	Non-Saudi	33	22
Smoking	Smoker	54	36
	Nonsmoker	96	64
Position	Resident	60	40
	Specialist	42	28
	Consultant	48	32
Sub-specialty	General Orthopaedics	66	44
	Sports	6	4
	Arthroplasty	12	8
	Paediatric Orthopaedics	12	8
	Spine	15	10
	Trauma	9	6
	Upper Limb	15	10
	Oncology	6	4
	Foot	6	4
	Pelvis	3	2

**Table 3:** Orthopedic surgeons’ mean, SD, minimum, and maximum scores from the SPADI scale.

SPADI Scores	Mean	Standard Deviation	Minimum	Maximum
Pain Components (= 5)				
At its worst? (0–10)	2.98	2.17	1.00	10.00
When lying on the involved side? (0–10)	2.52	1.91	1.00	8.00
Reaching for something on a high shelf? (0–10)	2.42	2.02	1.00	8.00
Touching the back of your neck? (0–10)	2.31	2.06	1.00	10.00
Pushing with the involved arm? (0–10)	2.44	1.96	1.00	10.00
PAIN SCORE (out of 50)	12.62	8.10	5.00	34.00
PAIN PERCENTAGE (%)	25.24	16.20	10.00	68.00
Disability Components (= 8)				
Washing your hair? (0–10)	2.00	1.80	1.00	10.00
Washing your back? (0–10)	2.22	1.83	1.00	9.00
Putting on an undershirt or jumper? (0–10)	2.10	1.82	1.00	10.00
Putting on a shirt that buttons down the front? (0–10)	1.94	1.65	1.00	10.00
Putting on your pants? (0–10)	1.78	1.68	1.00	10.00
Placing an object on a high shelf? (0–10)	2.18	1.87	1.00	10.00
Carrying a heavy object of 10 pounds? (0–10)	2.66	2.24	1.00	10.00
Removing something from your back pocket? (0–10)	2.12	1.91	1.00	10.00
**DISABILITY SCORE (out of 80)**	17.00	13.99	8.00	79.00
**DISABILITY PERCENTAGE (%)**	21.25	17.49	10.00	98.75
**Total SPADI components (= 13)**				
**SPADI SCORE (out of 130)**	29.62	21.02	13.00	113.00
**SPADI PERCENTAGE (%)**	22.79	16.17	10.00	86.92

**Table 4:** The correlation of various factors with the total SPADI percentages and their Spearman correlation Rho values.

Variables	Rho Value	*p*-Value
Marital Status	−0.199	0.166
Age in years	−0.113	0.436
Nationality	0.106	0.463
Smoking	−0.019	0.896
Position	−0.119	0.409
Years of experience	−0.038	0.796
Hours of work/day	0.052	0.72
Sub-specialty	−0.01	0.945
Height in meters	−0.266	0.061
Weight in kilograms	0.432 **	0.002
BMI in kilogram/meter square	0.349 *	0.013

** Correlation is significant at the 0.01 level (2-tailed). * Correlation is significant at the 0.05 level (2-tailed). Results were considered significant if the *p*-value was less than 0.05.

## Data Availability

Data are available from the corresponding author mentioned in this research paper.
